# Bioengineering strategies to enhance BCG immunotherapy against bladder cancer

**DOI:** 10.3389/fimmu.2026.1888889

**Published:** 2026-10-05

**Authors:** Senhao Li, Haige Chen, Bing Feng

**Affiliations:** 1 Department of Urology, Renji Hospital, Shanghai Jiao Tong University School of Medicine, Shanghai, China; 2 Zhejiang Cancer Hospital, Zhejiang Key Laboratory of Functional Nucleic Acids for Basic and Clinical Application, Hangzhou Institute of Medicine (HIM), Chinese Academy of Sciences, Hangzhou, Zhejiang, China

**Keywords:** BCG, bioengineering, bladder cancer, drug delivery, immunotherapy

## Abstract

Bacillus Calmette–Guérin (BCG) remains the frontline immunotherapy for non–muscle-invasive bladder cancer (NMIBC), yet recurrence and non-response occur in over half of patients, highlighting the need for improved therapeutic strategies. Recent advances in bioengineering including genetic modification, synthetic biology, biomaterials, and delivery systems are expanding BCG from a traditional vaccine into a more versatile immunotherapeutic platform. In this review, we summarize the mechanisms of BCG-induced antitumor immunity and discuss current bioengineering strategies aimed at enhancing tumor interaction, innate immune activation, and modulation of the tumor immune microenvironment (TIME). We further highlight emerging approaches in delivery system design to improve intravesical retention and therapeutic efficacy. In addition, we discuss evolving perspectives on immune regulation in BCG therapy, including the potential contribution of type 2 immunity alongside canonical type 1 responses, as well as the role of trained immunity in sustaining antitumor effects. Finally, we outline key challenges for clinical translation and future directions toward more rational and precise BCG-based immunotherapy.

## 1 Introduction

Bladder cancer ranks among the ten most prevalent malignancies worldwide, posing a persistent threat to global health. In 2022, approximately 610,000 new cases and 220,000 deaths were reported globally, and these numbers are projected to reach 990,000 and 400,000, respectively, by 2040 (1, 2). The treatment strategy for bladder cancer varies by stage and risk classification. NMIBC accounting for over 70% of initial diagnoses, is typically managed with transurethral resection of the bladder tumor, followed by intravesical instillation of BCG to prevent recurrence and progression (3–5). BCG, an attenuated strain of *Mycobacterium bovis* was first introduced for bladder cancer treatment by Morales in 1976 and approved by the U.S. Food and Drug Administration (FDA) in 1990 (6, 7). Beyond its historical role as a tuberculosis vaccine, BCG remains one of the most successful microbial immunotherapies in oncology, capable of eliciting durable tumor control through robust immune activation.

Despite its long-standing clinical success, it faces numerous challenges. A primary concern is the poor response rate. Approximately 40% of patients fail to respond to initial BCG treatment, and nearly half of responders eventually relapse or progress to more advanced disease. For these patients, radical cystectomy remains the standard therapeutic option. However, this surgery is highly invasive and significantly impacts quality of life, making it an intolerable choice for many elderly patients or those with comorbidities (8, 9). Moreover, BCG instillation can cause adverse local and systemic effects such as cystitis, bladder contracture, and fever, leading to treatment discontinuation in up to 8% of patients (10, 11). Compounding these issues, recurrent global shortages of BCG supply since 2012, further intensified by the COVID-19 pandemic have underscored the urgent need for alternative or improved formulations (12).

Recent advances in bioengineering spanning genetic engineering, synthetic biology, biomaterials, and advanced delivery systems, have opened new avenues for revitalizing BCG therapy. Recombinant BCG strains engineered to express immunostimulatory molecules have demonstrated enhanced immune activation and improved tumor selectivity. Concurrently, biomaterial-assisted and advanced delivery strategies enable prolonged bladder retention, targeted delivery, and reduced toxicity. In this review, we summarize the immunological mechanisms underlying BCG-induced antitumor responses and highlight emerging bioengineering strategies to improve its efficacy, precision, and safety. Particular attention is given to genetically modified BCG strains, biomaterial-assisted delivery systems, and hybrid synthetic biology approaches. Although BCG has been used clinically for decades, a unified framework integrating its immunological mechanisms with emerging bioengineering strategies remains lacking. Here, we aim to bridge this gap by systematically linking immune processes with engineering interventions to guide the rational design of next-generation BCG-based immunotherapies.

## 2 Mechanisms of BCG-induced antitumor immunity

The therapeutic efficacy of BCG in bladder cancer arises from its direct engagement with tumor cells and the subsequent activation of innate and adaptive immunity. BCG initially adheres to bladder cancer cells via both electrostatic interactions and receptor-mediated mechanisms, notably through the fibronectin attachment protein (FAP) binding integrin α5β1 on tumor cells (13, 14). Once bound, BCG is internalized via phagocytosis or macropinocytosis, ultimately fusing with lysosomes to form phagolysosome (**Figure 1**) (15). This internalization triggers direct tumoricidal effects, including inhibition of proliferation through cyclin D1/E downregulation (16), induction of apoptosis via caspase-dependent and mitochondrial pathways (17, 18), and generation of oxidative stress through inducible nitric oxide synthase (iNOS) and H_2_O_2_ production (19). Caspase-independent cell death further releases high mobility group box-1 protein (HMGB1), a hallmark of immunogenic cell death (ICD) that activates antigen-presenting cells (APCs) (20).

**Figure 1:**
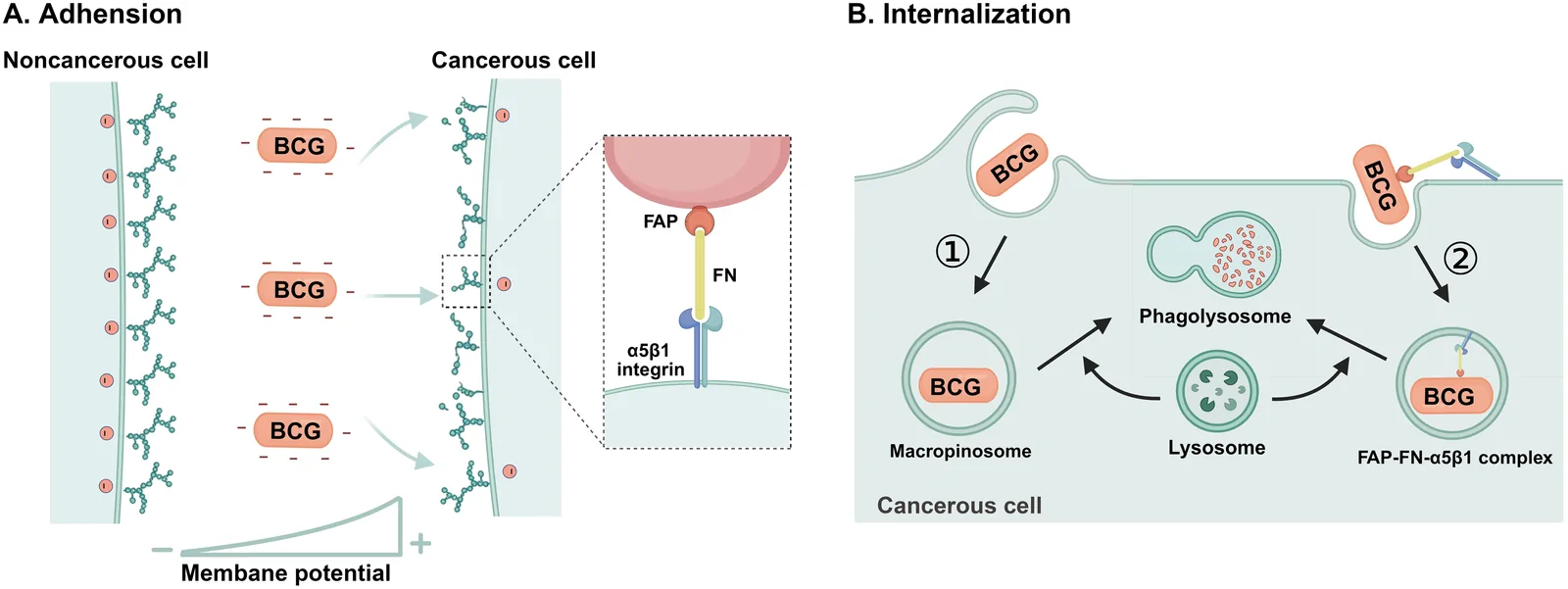
The adhesion and internalization of BCG. **(A)** This adhesion is primarily regulated by physicochemical mechanism and ligand-dependent mechanism. On one hand, cancer cells exhibit a less negatively charged surface due to deficiencies in the glycosaminoglycan layer, thereby reducing the electrostatic repulsion between these cells and BCG. On the other hand, the fibronectin attachment protein on the surface of BCG binds to cancer cell integrin α5β1 via fibronectin; **(B)** After adhering to cancer cells, BCG is internalized through two pathways: one is a macropinocytosis pathway, and the other is a receptor-mediated endocytosis guided by the FAP–FN–integrin α5β1 complex. Regardless of the pathway, internalized BCG will fuse with lysosomes to form phagolysosomes.

BCG’s antitumor activity is further amplified by robust innate immune activation. Pathogen-associated molecular patterns (PAMPs) on the BCG cell wall engage TLR on tumor cells and APCs, promoting cytokine secretion and upregulating major histocompatibility complex class I (MHC-I) on tumor cells, enhancing their antigen-presenting capacity (13, 21, 22). DCs as key APCs bridging innate and adaptive immunity, are central to BCG-mediated immune activation. BCG engages pattern recognition receptors (PRRs) such as DC-SIGN on DCs, activating the NF-κB pathway and upregulating anti-apoptotic factors including Bcl-2 and Bcl-xL, thereby enhancing DC survival and long-term immune activity (23). Among these, cDC1 is a key cross-presenting subset that specializes in presenting exogenous tumor antigens to CD8^+^ T cells via MHC-I, thereby inducing CTL responses. The absence of cDC1 renders intravesical bacterial immunotherapy ineffective and blocks CD8^+^ T cell infiltration (24). cDC2, in contrast, is biased toward MHC-II-mediated CD4^+^ T cell activation, promoting Th1/Th17-like differentiation and helper T cell responses, consistent with the local and systemic CD4^+^ Th1 responses observed after BCG treatment (25, 26). pDCs recognize mycobacterial DNA through TLR9, produce type I interferons, and participate in early innate immunity (27, 28); however, their ability to induce CD8^+^ T cell differentiation is weaker than that of cDC1 (29), and in the TME they are affected by Treg co-localization and inhibitory mechanisms such as PD-L1/IDO (30), so their function is often restricted or tends toward tolerance. mo-DCs can supplement local antigen uptake, presentation, and effector T cell re-stimulation in the context of BCG-induced inflammation and myeloid reprogramming; this is consistent with the mechanism by which BCG can reshape monocyte-myeloid cell function through trained immunity and enhance myeloid antitumor immunity (31). mregDCs (especially LAMP3^+^ DCs) respond to the CCL19/CCL21 chemokine axis via CCR7, migrate to lymphoid areas, and participate in T cell priming and tertiary lymphoid structure (TLS) formation (32). Mature follicular-like TLS and memory B cell enrichment can predict BCG treatment response and longer recurrence-free survival (33).

Beyond the DC-centered mechanisms described above, BCG also mobilizes a broader network of innate immune effectors—including macrophages, NK cells, neutrophils, γδ T cells, and mucosal-associated invariant T (MAIT) cells—that cooperate to amplify its antitumor activity. BCG reprograms macrophages toward an antitumor phenotype by upregulating M1-associated markers (TNF-α, CD86, iNOS) while suppressing M2 markers (IL-10, CD206, Arginase-1). Notably, BCG downregulates the anti-apoptotic protein Mcl-1, which not only reinforces M1 polarization but also promotes tumor cell apoptosis through intercellular communication mediated by connexin 43 (Cx43) (34). The cytotoxic activity of BCG-activated peripheral blood mononuclear cells (PBMCs) is mainly mediated by CD56^+^/CD3^-^NK cells (35). Nevertheless, BCG alone cannot directly activate purified NK cells and its immunostimulatory effect requires cooperation with other immune cells (36). Monocytes activated by BCG release IL-12 and IFN-α, which in turn stimulate NK cells to produce IFN-γ and exert cytotoxicity through the perforin–granzyme pathway. Notably, BCG treatment does not significantly alter NK cell counts in patients but instead boosts their functional activity, primarily through NKG2D-dependent mechanisms (36, 37). Neutrophils are indispensable mediators of BCG-induced antitumor immunity, as their depletion completely abolishes the therapeutic efficacy of BCG therapy and combination regimens (38). BCG-activated neutrophils exert cytotoxicity through neutrophil extracellular traps (NETs) formation and TNF-related apoptosis-inducing ligand (TRAIL) secretion. Neutrophils can shape the TIME by releasing cytokines and chemokines such as IL-1, IL-6, IL-8, IL-12, TNF-α, MIP-2, IP-10, and MIG, thereby amplifying local immune activation (39). γδ T cells, a unique subset bridging innate and adaptive immunity, also play a crucial role in BCG-induced anti-tumor responses. Unlike conventional T cells, they recognize diverse antigens independently of MHC molecules and can prime effector T cells either directly or by enhancing dendritic cell function (40, 41). BCG selectively expands anti-tumor γδ T-cell subsets, particularly Vγ9Vδ2 and Vγ8Vδ2, which express markers such as CD27 and NKG2D, exhibit strong cytotoxicity, and secrete IFN-γ. These cells kill bladder cancer cells via perforin/granzyme, TRAIL, and FasL/Fas pathways, and serve as a major source of local IL-17, essential for BCG-induced neutrophil recruitment (42). Importantly, γδ T-cell expansion requires interactions with other immune cells and is not induced by BCG alone (37, 43). Although less abundant in peripheral blood, MAIT cells produce high levels of cytokines upon BCG stimulation and contribute to anti-tumor activity. In bladder cancer patients, the proportion of IFN-γ–producing MAIT cells correlates with tumor burden and invasiveness, highlighting their potential role in BCG-mediated immunotherapy (43, 44).

A crucial component of BCG-induced antitumor activity is trained immunity, or called innate immune memory, which mediates long-term, non-specific protection by epigenetic and metabolic reprogramming of innate immune cells (45). Circulating monocytes isolated from NMIBC patients receiving BCG treatment exhibit enhanced TNF and IL-1β production upon heterologous ex vivo stimulation (46). Clinical evidence shows that systemically acquired trained immunity in circulating monocytes correlates with long-term recurrence-free survival in NMIBC patients following BCG therapy (47). A recent study using a mouse model of *in situ* bladder cancer and peripheral blood samples from NMIBC patients revealed the mechanism by which BCG induces trained immunity. BCG can reach the bone marrow, where it reprograms hematopoietic stem and progenitor cells (HSPCs) via interferon regulatory factor (IRF) and signal transducer and activator of transcription (STAT) transcription factors. This generates downstream myeloid cells including neutrophils, monocytes, and DCs with enhanced effector functions (48). Trained neutrophils show increased ROS production, tumor infiltration, anti-angiogenic activity, and rapid TRAIL secretion upon stimulation, while monocytes and dendritic cells display improved antigen presentation, T cell activation, and resistance to immunosuppressive signals (38, 49). The induction and magnitude of trained immunity are influenced by BCG strain and host autophagy status. A mouse intravenous inoculation model showed that BCG-China and BCG-Pasteur had a stronger capacity to induce trained immunity, whereas BCG-Russia was weaker (50). By combining *in vitro* experiments with human monocytes and follow-up of patients with NMIBC patients who received intravesical BCG, the study found that autophagy drives BCG/β-glucan-induced monocyte trained immunity by promoting H3K4me3 epigenetic reprogramming; polymorphisms in autophagy-related genes such as ATG2B and ATG5 can attenuate this training effect and are associated with the risk of recurrence and progression after intravesical BCG therapy in NMIBC patients (51). Furthermore, BCG can be combined with other trained immunity inducers; for example, co-administration with fungal-derived β-glucan synergistically reprograms the granulopoietic trajectory of HSPCs, generating “trained neutrophils” with potent and durable anticancer capacity. Significant antitumor effects were observed in both mouse bladder cancer and melanoma models (49).

The adaptive immune response consolidates innate immunity. BCG activates CD4^+^ T cells via APCs, promoting their differentiation toward Th1 cells, which secrete IFN-γ and IL-2 (52). CD4^+^ T cells may also polarize toward a Th17 phenotype, yet the functional implications of the Th17/IL-17 axis in bladder cancer are complex and context-dependent. Increased Th17 and IL-17 levels have been linked to aggressive tumor features, including high grade, lymph node metastasis, and angiogenesis, implying tumor-promoting potential (53–55). Conversely, IL-17-driven neutrophil recruitment may augment antitumor immunity, and elevated IL-17^+^ cell infiltration has been associated with improved responses to BCG therapy (42). IL-17 plays a distinct yet paradoxical dual role in tumor immunity. Pro-tumor effects typically manifest during early stages of tumorigenesis, in specific mutational contexts (such as Kras-driven lung cancer and APC-mutation-associated colorectal cancer), and within chronic inflammatory microenvironments: in these settings, IL-17 promotes cancer cell proliferation, survival, and epithelial-mesenchymal transition by directly activating intracellular signaling pathways such as NF-κB, MAPK, and STAT3; simultaneously promoting angiogenesis by inducing VEGF and recruiting immunosuppressive cells such as MDSCs and tumor-associated neutrophils, thereby creating an immunosuppressive microenvironment that accelerates tumor growth and metastasis—a phenomenon clinically associated with poor prognosis. Antitumor effects, however, are more commonly observed in established tumors or environments with favorable immunological conditions: When Th17 cells differentiate into a Th1-like phenotype under the influence of cytokines such as IL-12 and IL-1β and co-express IFN-γ, IL-17 can effectively recruit dendritic cells, CD8^+^ cytotoxic T cells, and NK cells to the tumor site, activating an antitumor immune response and even directly inducing tumor cell apoptosis; In certain models (such as melanoma and some lung cancer models), overexpression of IL-17 can actually inhibit tumor growth and metastasis and is associated with a better prognosis. Therefore, whether IL-17 is an “enemy” or an “ally” depends on the cytokine network in the TME, the polarization state of immune cells, and the stage of tumor development (56–58). In previous mouse models, BCG instillation was observed to mainly induce large-scale recruitment of CD4^+^ T cells to the bladder mucosa, with minimal changes in their transcriptional phenotype, leading to the preliminary conclusion that BCG primarily promotes CD4^+^ T-cell infiltration rather than sufficient functional activation (59). However, such inferences based on animal models cannot fully reflect the true TME of human patients. Several recent single-cell transcriptomic studies based on patient samples have revealed the existence of highly activated and cytotoxic CD4^+^ T cell populations in human bladder cancer (60), which can directly kill autologous tumor cells in an MHC-II-dependent manner (61). The local activation of BCG-specific T cells themselves also plays a crucial role in the therapeutic mechanism. A longitudinal cohort study covering 32 high-risk NMIBC patients showed that most BCG-untreated patients already had pre-existing BCG-specific CD4^+^ T cells due to childhood BCG vaccination. After induction by BCG immunotherapy, the frequency and degree of memory differentiation of these specific cells in peripheral blood increased significantly, and they exhibited a multifunctional Th1 phenotype. Importantly, these BCG-specific CD4^+^ Th1 cells could also be detected in the urine of patients during treatment, demonstrating that they were successfully recruited to the local bladder and responded strongly to intravesical BCG stimulation by producing inflammatory cytokines (25). While classical theory emphasizes that Th1 immunity is essential for BCG’s antitumor effect and that a Th2 orientation may be detrimental, recent clinical evidence challenges this viewpoint. In a cohort of 23 high-risk NMIBC patients, a pre-treatment Th2 bias—defined by a high GATA3/T-bet ratio—predicted stronger Th1 responses following BCG therapy, suggesting that a Th2-biased microenvironment can prime and enhance Th1 activation (62). Both *in vitro* and *in vivo* studies further support this concept, showing IL-4, while inducing macrophage polarization toward the classical M2 type, can also mediate a non-classical M2INF macrophage with potent pro-inflammatory potential through the glycolysis/HIF-1α axis and Wdr5-mediated H3K4me3 epigenetic modification. These cells, when subsequently stimulated by LPS or pathogens, enhance production of inflammatory cytokines and NO, while also boosting resistance to bacterial infections. This suggests IL-4 may function as a β-glucan-like inducer of trained immunity, reshaping macrophage metabolism and immune function (63). Additionally, IL-4 can inhibit tumor growth and synergizes with TNF-α to induce apoptosis, underscoring the potential of Th2 signals to augment BCG-induced Th1 immunity (62).

CD8^+^ T cells are key cytotoxic effectors in BCG-mediated antitumor responses. Traditionally, their activation was attributed to cross-talk with CD4^+^ T cells, macrophages, and APCs (21), but recent studies show that BCG can also directly promote CD8^+^ T cell proliferation and activation via upregulation of FLT3 ligand (FLT3LG) through the FLT3/FLT3LG pathway (64). Multi-omics analyses reveal that responding tumors are enriched in activated PD-1^-^CD8^+^ T cells, whereas non-responders predominantly harbor exhausted PD-1^+^CD8^+^ T cells, establishing a direct link between CD8^+^ T cell states and clinical outcomes (65, 66). Notably, the spatial distribution of CD8^+^ T cells—specifically the ratio of epithelial to stromal densities—predicts recurrence-free survival more effectively than absolute cell densities, emphasizing the importance of tissue context in antitumor immunity (67).

Beyond these cell-specific mechanisms, the local immune response to BCG also evolves over time and differs between bladder tissue and urine. In the early phase (hours to days after the first instillation), bladder tissue is characterized by massive neutrophil infiltration (68, 69). However, urine observations show that urothelial chemokine release, including an early peak in IL-8, precedes a prominent neutrophilic influx during the first hours to days after instillation (70). About 24 hours after instillation, neutrophils account for 65%–80% of urinary leukocytes; although monocytes/macrophages and T cells are recruited, their abundance is low (6). During multiple instillations, bladder tissue develops chronic granuloma-like infiltration, dominated by monocytes/macrophages, T cells, and NK cells, with a marked increase in CD4^+^ T cells (71). Urine is still dominated by activated neutrophils, with very few lymphocytes; neutrophil activation is directly correlated with the amount of urothelial shedding (72, 73). In the late and maintenance phases, the tissue immune landscape diverges and determines prognosis: responders are dominated by activated CD8^+^PD-1^-^ T cells and non-Treg CD4^+^FOXP3^-^ effector T cells, maintaining durable immune surveillance (65);in non-responders, effector T cells return to pretreatment baseline, and the microenvironment becomes dominated by FoxP3^+^ Tregs and M2 macrophages, showing an immunosuppressive or tumor-promoting state (71, 74). These observations should be interpreted according to the sampling compartment: bladder tissue preserves spatial and tissue-resident immune features, whereas urine enables minimally invasive longitudinal monitoring of cells and soluble mediators released into the bladder lumen. Recent single-cell analysis further showed that urine-derived immune cells more closely resemble tumor-infiltrating immune cells than peripheral-blood immune cells, supporting their use as a complementary, but not equivalent, proxy for local immune responses (60).

In summary, BCG-induced antitumor immunity is not the result of isolated or simultaneous activation of individual immune-cell populations, but rather a coordinated and temporally evolving process involving tumor engagement, direct cytotoxicity, innate immune activation, trained immunity, and adaptive T-cell responses. Its therapeutic efficacy is further shaped by immune-cell polarization, spatial distribution, and intercellular crosstalk. This integrated mechanistic framework provides the rationale for the engineering strategies discussed in the following sections, which aim to enhance the immunogenicity, precision, and safety of BCG-based therapy (**Figure 2**).

**Figure 2:**
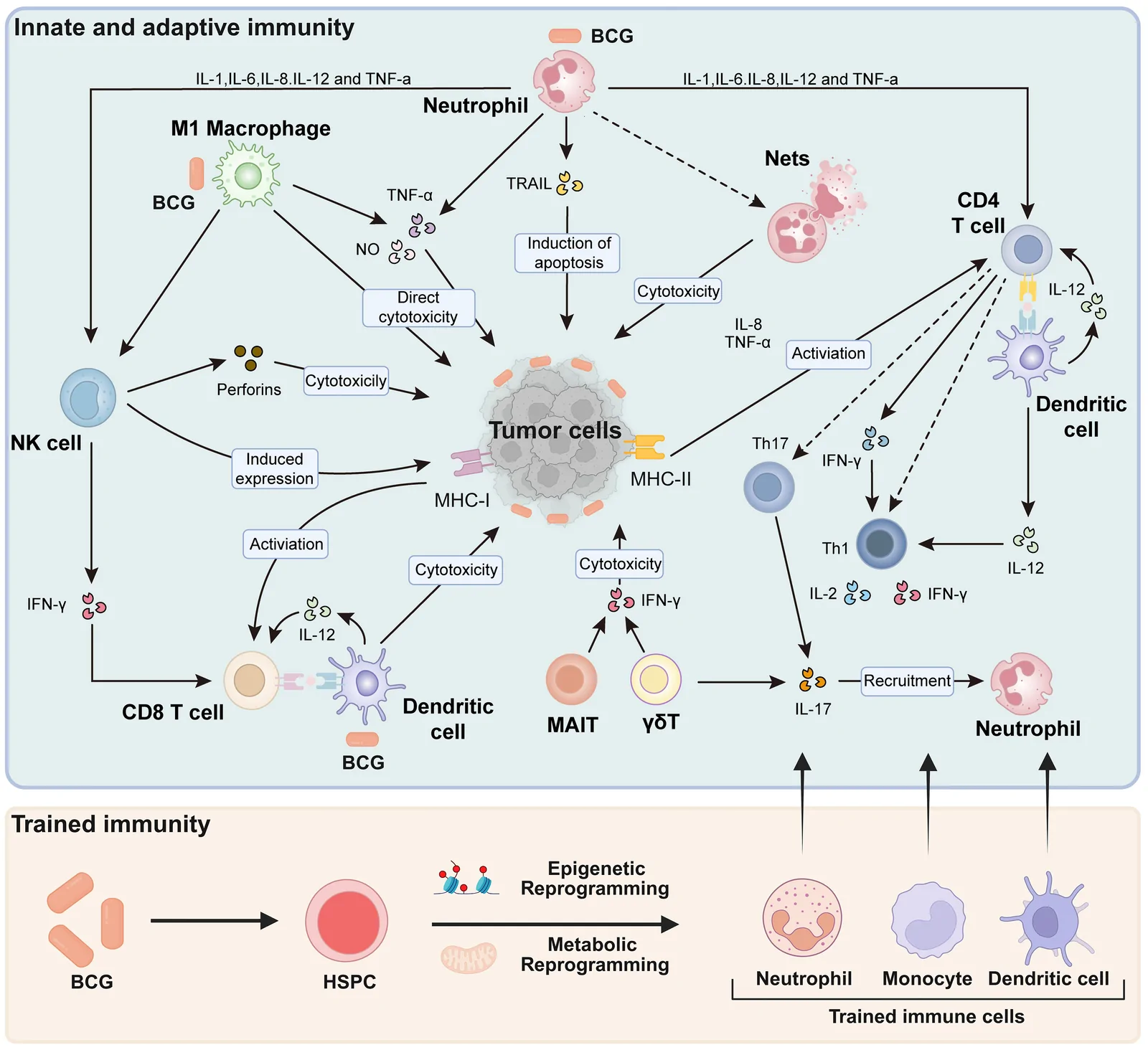
The immune response activated by BCG. BCG activates both innate and adaptive immune responses. Macrophages are reprogrammed into the M1 phenotype and downregulate Mcl-1 to promote apoptosis; NK cells are activated by monocyte-derived IL-12/IFN-α and exert cytotoxic effects via the perforin-granzyme pathway; neutrophils form NETs and secrete TRAIL, serving as indispensable mediators of therapeutic efficacy; DCs activate the NF-κB pathway and secrete IL-12 to induce Th1 polarization; γδ T cells and MAIT cells kill tumors in a non-MHC-restricted manner and secrete IFN-γ. In adaptive immunity, CD4^+^ T cells differentiate into Th1 cells that secrete IFN-γ and IL-2, while CD8^+^ T cells act as the primary cytotoxic effector cells and directly eliminate tumors. This process also involves monocytes, DCs, and neutrophils that have been reprogrammed via BCG colonizing in the bone marrow.

## 3 Engineering strategies to enhance BCG-mediated antitumor immunity

As discussed above, the antitumor efficacy of BCG relies on a coordinated cascade of tumor interaction, antigen presentation, innate immune activation, trained immunity, and adaptive T cell responses. However, each of these processes is subject to intrinsic limitations, including insufficient tumor–BCG contact, suboptimal antigen presentation, inadequate immune activation, and rapid drug clearance within the bladder. To address these challenges, recent bioengineering strategies have been developed to enhance specific steps of the BCG-mediated immune response. These approaches can be broadly categorized into four functional aspects: improving tumor interaction and antigen presentation, enhancing innate immune activation and trained immunity, reprogramming the TIME, and optimizing delivery efficiency and intravesical retention.

### 3.1 Improving tumor interaction and antigen presentation

Efficient interaction between BCG and tumor cells, as well as effective antigen processing and presentation, are critical for initiating robust downstream immune responses. Strategies that enhance bacterial adhesion, persistence, and antigen accessibility can therefore significantly improve therapeutic outcomes.

#### 3.1.1 Bioadhesion-mediated enhancement of bladder colonization

Following intravesical BCG instillation, short bladder retention from urinary washout and voiding restricts effective BCG-tumor contact. Insufficient adherence may contribute to treatment failure in some patients (75–77). Therefore, strengthening BCG’s attachment to the urothelium is expected to improve its therapeutic efficacy. It is worth noting that uropathogenic *Escherichia coli* relies on its adhesin FimH to bind mannose residues on the urothelial cell surface, thereby achieving efficient colonization. Beyond its natural function as an adhesin, FimH can also serve as an immune adjuvant in cancer immunotherapy (78, 79).Based on this mechanism, the recombinant strain (rBCG-S.FimH) overexpressing FimH is developed (80).It demonstrated significantly enhanced adhesion and internalization by urothelial cells, as well as prolonged retention in the bladder. Functionally, rBCG-S.FimH augmented the antigen-presenting capacity of DCs and the Th1 immune response. As BCG itself acts as an immune adjuvant, its functionality was further potentiated by the co-expression of FimH. While rBCG-S.FimH exhibits enhanced adhesion and immune activation, its attachment to bladder urothelium lacks specificity—binding both tumor and normal urothelial cells. To improve targeting precision, future strategies may involve fusing FimH with tumor-specific targeting molecules, such as antibodies or peptides. From a translational perspective, stratifying patients based on mannose expression levels in tumor tissues may help assess their response to BCG instillation therapy (**Figure 3A**).

**Figure 3:**
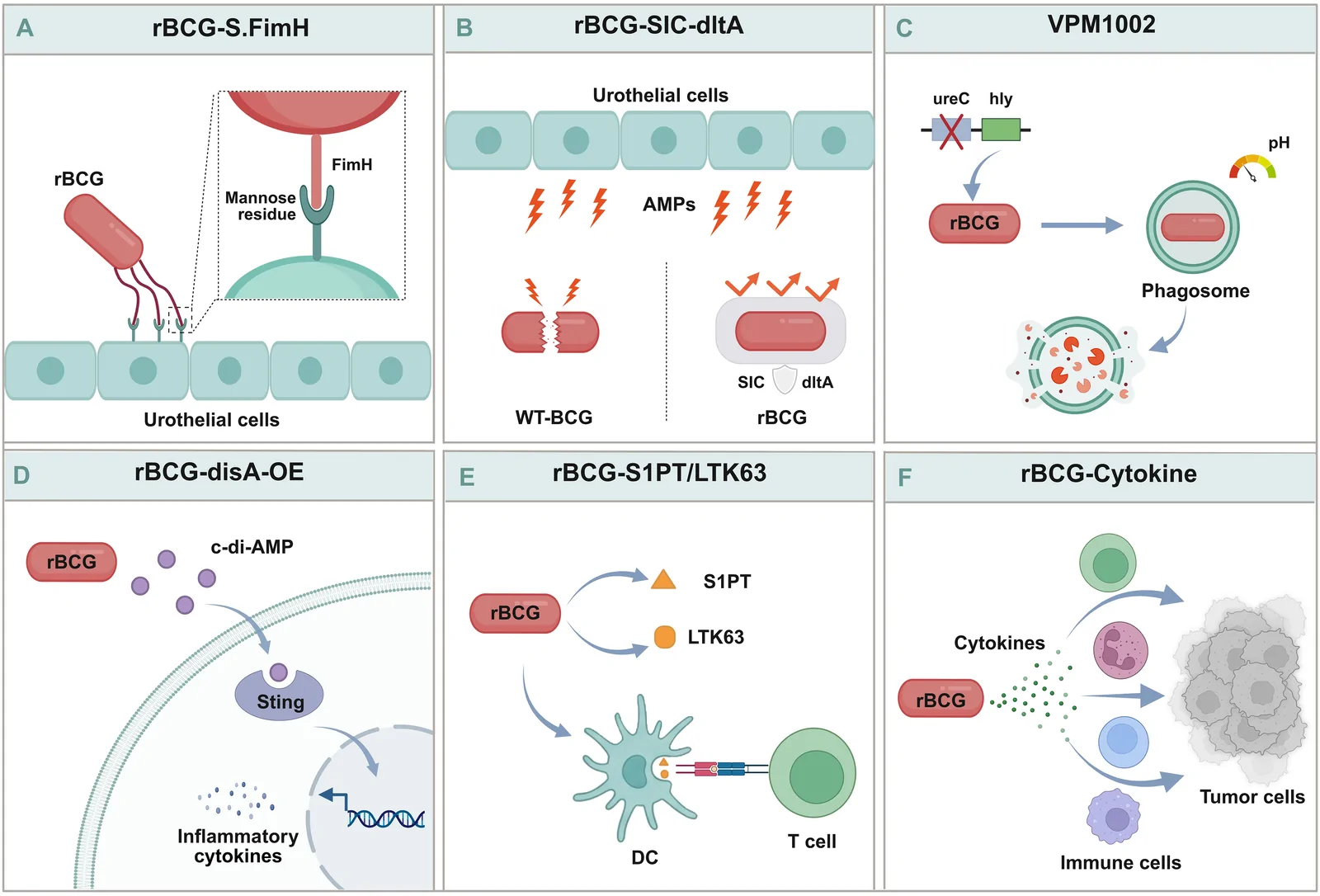
BCG’s engineering strategies. **(A)** rBCG-S.FimH was constructed for stable FimH expression, which markedly improved mucosal adhesion and retention through high-affinity binding to host mannose residues; **(B)** rBCG-SIC-dltA counteracts the AMPs secreted by urothelial cells by secreting SIC and dltA, potentiating its survival, internalization, and immunostimulation in bladder cancer cells; **(C)** By deleting the ureC gene in VPM1002 and inserting the hly gene, pores are formed in the phagosome membrane, releasing more antigenic substances; **(D)** rBCG-disA-OE was constructed by placing disA under a strong promoter to drive c-di-AMP overexpression, thereby activating the STING pathway and subsequent inflammatory responses; **(E)** rBCG-S1PT/LTK63 was engineered to stably express a detoxified heterologous toxin, leveraging its potent adjuvant properties to enhance the BCG-induced immune response; **(F) **rBCG-Cytokines enhance the immune response by secreting various cytokines.

#### 3.1.2 Exogenous protein-enhanced resistance to host antimicrobial peptides

When bacterium acts on the bladder, urothelial cells secrete AMPs, thereby activating the host’s immune defense system to clear the bacterium (81). However, certain cells can evade this AMPs-mediated killing by secreting specific proteins, such as streptococcal inhibitor of complement (SIC) and the D-alanyl carrier protein ligase (dltA) (82, 83). The suboptimal efficacy of BCG in some patients may be attributed to the effect of AMPs on BCG (84). To overcome this limitation, the recombinant BCG strain expressing SIC and dltA is constructed (85–87). This rBCG exhibits enhanced resistance to AMPs and promotes macrophage migration in a concentration-dependent manner. In addition, it demonstrates higher internalization efficiency, and cancer cells infected with rBCG significantly upregulate the expression of IL-6, IL-12, TNF-α, and IFN-γ. The antitumor efficacy of this rBCG was validated in mouse models. Enhancing BCG resistance to AMPs may improve bacterial persistence and promote macrophage recruitment and activation, potentially involving trained immunity through myeloid reprogramming. Future studies should clarify these mechanisms and validate the efficacy and safety of this strategy in clinical settings. In conclusion, leveraging the natural mechanism by which bacteria evade host antimicrobial peptide clearance to enhance the *in vivo* activity of BCG offers a new strategy for improving its anti-tumor efficacy (**Figure 3B**).

#### 3.1.3 Genetic replacement-enhanced immunogenicity

The wild-type BCG, due to the absence of the ESX-1(ESAT-6 secretion system-1), remains confined within the phagosome after internalization. As a result, its antigens cannot effectively enter the antigen presentation pathway, leading to a weak CD8^+^ T cell response and limited antitumor efficacy (88, 89). Therefore, enhancing effective antigen presentation is expected to improve the therapeutic effect of BCG. VPM1002 is a recombinant genetically modified vaccine based on BCG, engineered through the deletion of the urease C gene (ureC) in BCG and the insertion of the listeriolysin O encoding gene (hly) from *Listeria*. This genetic modification enables the phagosome containing the vaccine to acidify rapidly upon entering host cells, providing an optimal acidic environment for listeriolysin O(LLO) stability. Subsequently, LLO forms pores in the phagosomal membrane, facilitating the release of mycobacterial antigens and DNA into the cytosol, thereby effectively triggering robust immune responses (90, 91). VPM1002 has demonstrated a favorable immunogenicity and safety profile in mammalian models. It not only effectively activates Th1 response but also induces a more potent IFN-γ response in CD4^+^ T cells compared to BCG, all without eliciting systemic health concerns (92). A Phase 1/2 clinical trial evaluating VPM1002 for the treatment of patients with NMIBC who relapsed after prior standard BCG therapy has been completed. Results demonstrated encouraging efficacy and safety of VPM1002 in this high-risk population. Among the 40 enrolled patients, the bladder recurrence-free survival rate was 49.3% at 60 weeks and remained at 43.7% at 3 years. Treatment-related adverse events were predominantly mild to moderate, with no Grade ≥4 events reported, indicating good tolerability (93). Although VPM1002 enhances antigenic cross-presentation through LLO-mediated pore formation, persistent damage to the phagosome membrane may induce excessive autophagy or cytoplasmic DNA leakage, triggering abnormal inflammatory responses or even autoimmune risks. On the one hand, LLO forms pores in the phagosome membrane, facilitating bacterial escape into the cytoplasm and inducing a stress response, which in turn activates autophagy—it not only regulates the expression of the autophagy adaptor protein Optineurin to mediate the binding of ubiquitinated bacteria to microtubule - associated protein light chain 3(LC3), but also promotes LC3-associated phagocytosis by triggering membrane damage signals, thereby enhancing immunity against Listeria (94–96); on the other hand, LLO may also inhibit autophagy; for example, bacterial-derived membrane vesicles carrying oxidatively inactivated LLO can block autophagic flux and hinder cell death, thereby promoting bacterial survival (94). This fine-tuned regulation suggests that sustained expression of LLO in vaccine vectors or its leakage into the cytoplasm may disrupt autophagic homeostasis, and both autophagic dysfunction and excessive activation are closely associated with autoimmune diseases (97, 98). Furthermore, LLO-induced changes in membrane permeability potently activate the NLRP3 inflammasome, leading to massive release of IL-1β and IL-18. This process is not only a key component of the anti-infective immune response but also a core pathological mechanism underlying autoimmune diseases such as systemic lupus erythematosus and rheumatoid arthritis (99). Although there is currently no direct evidence that LLO induces classic autoimmune diseases through molecular mimicry, the intense inflammatory environment it induces, epitope spreading, and the potential for bystander activation as a potent inducer of CD8^+^ T cells still pose a small risk of triggering autoimmune responses in genetically susceptible individuals (100, 101). Furthermore, more than half of patients experience recurrence within three years, indicating that the therapeutic benefit of this vaccine is limited for populations that are already resistant to or unresponsive to BCG; combination with immune checkpoint inhibitors or chemotherapy drugs is expected to enhance its efficacy (**Figure 3C**).

### 3.2 Enhancing innate immune activation and trained immunity

Effective BCG therapy relies on robust activation of innate immune responses and the establishment of trained immunity to sustain long-term antitumor effects. Therefore, engineering strategies that amplify innate signaling pathways and metabolic reprogramming have emerged as key approaches to improve BCG efficacy.

#### 3.2.1 STING-targeted enhancement of innate and trained immunity

The STING pathway is a key focus in cancer immunotherapy for driving type I interferon and NF-κB-mediated antitumor immunity. Upon activation, IFN-β and pro-inflammatory cytokines are produced to enhance APCs maturation and cross-presentation, thereby activating tumor-specific CD8^+^ T cells and inhibiting tumor angiogenesis (102–105). Given that the STING pathway plays a critical role in the antitumor immune response induced by BCG (106, 107), combining BCG with STING agonists represent a promising strategy to enhance its therapeutic efficacy. Therefore, a recombinant BCG strain (BCG-disA-OE) was engineered to achieve high-level cyclic di-AMP (c-di-AMP) production by fusing the endogenous diadenylate cyclase gene (disA) with a constitutive promoter, markedly potentiating Sting activation (108). BCG-disA-OE promoted greater release of pro-inflammatory cytokines, driving macrophages toward the M1 phenotype and enhanced their phagocytic function. It also triggers upregulation of the glucose transporter 1(GLUT1) to enhance glycolysis in macrophages while reducing the immunosuppressive metabolite kynurenine. Macrophages stimulated by BCG-disA-OE produced significantly higher levels of itaconate, a metabolite that inhibits bacterial energy metabolism; Since BCG is a live bacterial therapy, the accumulation of itaconic acid may exert an antibacterial effect by inhibiting isocitrate lyase and succinate dehydrogenase, thereby limiting the survival time of BCG-disA-OE in the bladder and consequently shortening the window of immune stimulation. *In vitro* experiments have shown that itaconic acid exhibits antibacterial effects at concentrations as low as 24 mM (109). Although bladder urine is continuously diluted and drained, it is entirely possible to achieve effective antibacterial concentrations within the local microenvironment of the bladder wall mucosa. The impact of accumulated itaconic acid on BCG could be mitigated by genetically engineering BCG to overexpress itaconic acid metabolizing enzymes or by employing an intermittent dosing strategy. Furthermore, existing BCG therapies (such as bladder instillation) inherently require bacterial clearance within a few weeks to prevent disseminated infection. The enhanced immunostimulatory capacity of BCG-disA-OE may enable it to achieve equivalent efficacy at lower doses and with shorter treatment durations, while itaconic acid-mediated self-inhibition serves as a natural safety valve, preventing excessive inflammation and the formation of residual bacteria. In summary, future research must precisely elucidate the thresholds of itaconic acid’s bidirectional regulation of the BCG metabolic-immune axis to design an optimized live-virus vector that can both avoid premature clearance and prevent uncontrolled proliferation. Although both BCG-disA-OE and WT-BCG enhanced intratumoral CD4^+^and CD8^+^ T cell recruitment, only BCG-disA-OE increased splenic CD8^+^ T cell accumulation, indicating systemic reshaping of the T cell landscape—a distinct advantage likely contributing to more durable antitumor immunity. Notably, despite eliciting stronger inflammatory responses, BCG-disA-OE exhibited lower pathogenicity in animal models, supporting a favorable safety profile alongside its enhanced antitumor efficacy. By precisely “training” the immune system to attack tumors through enhanced Sting signaling while limiting collateral damage, BCG-disA-OE offers a promising strategy to dissociate efficacy from toxicity in immunotherapy (**Figure 3D**).

#### 3.2.2 Toxin antigens-mediated expansion of immune responses

Several detoxified bacterial toxin derivatives, such as the S1 subunit of pertussis toxin (S1PT), tetanus toxin fragment C (FC), and the non-toxic A subunit of the heat-labile toxin (LTK63), are widely investigated in vaccines for various diseases due to their low toxicity and high immunogenicity, demonstrating promising immune-inducing effects in animal models (110, 111).To bolster immune efficacy against bladder cancer, a recombinant BCG strain (rBCG-S1PT) that expresses S1PT was engineered. In human blood, rBCG-S1PT increased IL-8, IL-6, and IL-10 without elevating IL-1β or TNF-α, indicating a modified inflammatory profile. WT-BCG significantly raised IFN-γ, while rBCG-S1PT showed only a trend. Cytokine responses were 5–10 times higher in BCG-vaccinated than in BCG-naïve individuals, though both remained within the same order of magnitude. rBCG-S1PT upregulated CD25 and CD69 on CD4+ T cells, with slightly weaker CD69 induction than WT-BCG. rBCG-S1PT-primed PBMCs showed potent and dose-dependent cytotoxicity against bladder cancer cells, reaching nearly 100% at high effector-to-target ratios (112). A similar study also confirmed the potential of rBCG-S1PT in enhancing antitumor immune responses (113). Similarly, rBCG-LTAK63 (expressing LTK63) displayed stronger antitumor activity than conventional BCG. It enhanced DCs and T cell activation, promoted immune infiltration, and reduced regulatory T cells by ~50%. In mouse bladder cancer models, it significantly inhibited tumor growth and prolonged survival, demonstrating superior immunotherapeutic potential (114). While rBCG-S1PT upregulates IL-8, IL-6, and IL-10 but does not elevate IL-1β or TNF-α, it remains unclear whether this “modified” inflammatory response truly benefits antitumor activity or may instead weaken immune surveillance. The lack of a significant increase in IFN-γ is also concerning, as IFN-γ is a key factor in the antitumor Th1 response. Although toxin derivatives are theoretically low-toxicity, long-term follow-up is still needed to determine whether repeated bladder instillations induce local autoimmunity, chronic inflammation, or rare toxicities (**Figure 3E**).

### 3.3 Reprogramming the TIME

Beyond initial immune activation, the therapeutic efficacy of BCG is critically shaped by the TIME. Engineering strategies that modulate cytokine networks and immune cell functions can help establish a more favorable and sustained antitumor milieu.

#### 3.3.1 Exogenous cytokine-mediated remodeling of TIME

Following BCG instillation, massive cytokine release reshapes the TIME, directly recruiting and activating immune cells while modulating tumor cell invasion and migration through epithelial-mesenchymal transition (EMT)-related pathways (115–118). Some studies have focused on developing cytokine-secreting BCG strains, leveraging cytokine co-delivery to amplify antitumor efficacy. rBCG-IL-2 significantly enhanced the macrophage-mediated killing activity against cancer cells and promoted the production of TNF and IFN-γ. Furthermore, endogenously secreted IL-2 proved more efficient than exogenously supplied IL-2, achieving comparable effects at substantially lower concentrations (119). rBCG-IL-18 enhances IFN-γ production to an extent comparable to that achieved by combining BCG with exogenous IL-18. It also strengthens BCG-induced macrophage cytotoxicity against cancer cells in a dose-dependent manner, while demonstrating a favorable safety profile (120). rBCG-IL-15 drives neutrophil recruitment by upregulating the chemokines MIP-2 and MIP-1α, and its antitumor effect does not fully rely on classical IL-17^+^ γδ T cells, suggesting the existence of an alternative mobilization mechanism (121). rBCG-IFN-γ upregulates MHC-I expression on tumor cells and promotes the recruitment of CD4^+^T cells to the bladder. It prolongs mouse survival in a manner dependent on host endogenous IFN-γ at low doses, but no additional effect is observed at high doses and it unexpectedly induces IL-4 expression, suggesting that a Th2 response may be involved in its antitumor effect (122). rBCG-IFN-α can enhance the cytotoxicity of peripheral blood mononuclear cells (with NK cells as the primary effector cells), but it has a narrow effective dose range and requires precise control (123). The rBCG co-expressing IFN-α and GM-CSF can form a broader immune synergistic network, recruiting T cells while inducing macrophage aggregation, and upregulating key immune genes such as *Ccr2*, *Icos*, and *Tbx21*, thereby further enhancing therapeutic efficacy (124). While current strategies predominantly focus on type 1 cytokines to enhance BCG efficacy, emerging evidence suggests that this paradigm may be overly restrictive. The induction of IL-4 in rBCG-IFN-γ-treated settings implies that type 2 immunity could also contribute to antitumor effects, potentially by supporting immune cell function and modulating the TIME. Therefore, future studies should move beyond single-cytokine optimization and instead explore rational combinations of type 1 and type 2 cytokines, with an emphasis on their dynamic balance and context-dependent roles in shaping durable antitumor immunity (**Figure 3F**).

### 3.4 Combined application of engineering BCG and immune checkpoint inhibitors

Immune checkpoint inhibitors (anti-PD-1, anti-PD-L1, anti-CTLA-4) are integral to bladder cancer treatment. They block inhibitory receptor-ligand interactions (e.g., PD-1/PD-L1, CTLA-4/CD80, NKG2A/HLA-E, TIGIT/CD155) on T/NK cells, enabling immune recognition and attack of tumors (4, 125, 126). PD-1/PD-L1 inhibitors are most studied because BCG upregulates PD-L1 on bladder cancer cells, suppressing CTL activity and causing immune evasion and BCG resistance (127). Thus, combining BCG with anti-PD-(L)1 antibodies may overcome this immunosuppression and boost antitumor immunity. In an immunocompetent rat model, BCG+anti-PD-L1 outperformed monotherapy in reducing tumor growth and extending survival, enhancing antitumor immunity via increased tumor-infiltrating CD8^+^ T cell counts and function (granzyme B, IFN-γ, TNF-α) and reduced immunosuppressive MDSCs (127). This provides robust preclinical evidence for BCG-immune checkpoint inhibitor combinations. Multiple clinical studies have explored BCG plus PD-1/PD-L1 inhibitors. Combinations with durvalumab, pembrolizumab, or sasanlimab show favorable outcomes and synergy (128, 129). Results from a recent Phase III clinical trial indicate (130) that sasanlimab plus BCG (induction+maintenance) significantly improved event-free survival (36-month EFS: 82.1%), especially in carcinoma *in situ* or T1 tumors. Adverse events were higher but manageable. This study first demonstrated that sasanlimab requires full BCG induction and maintenance to improve efficacy; adding it to induction alone showed no advantage over standard BCG, underscoring the necessity of BCG maintenance in NMIBC.

The combination of BCG and PD-1/PD-L1 inhibitors is a key focus in high-risk NMIBC. Large Phase III trials, including KEYNOTE-676, are evaluating this strategy, with early results showing clinical benefit. Engineered BCG strategies—enhancing immune evasion, optimizing PAMP signaling, or surface modification for immunomodulator delivery—form a toolkit that boosts BCG’s immunostimulatory function for combination with ICIs. This synergy creates an organic network in two dimensions. First, bidirectional tumor immune microenvironment remodeling: BCG recruits and activates immune cells but also induces PD-L1/PD-1 expression, creating a negative feedback loop. ICIs release this brake, restoring and amplifying BCG-triggered antitumor immunity. Second, systemic immune coordination: BCG enters the bone marrow via mucosa-associated lymphoid tissue, reprogramming HSPCs and enhancing myeloid hematopoiesis. Engineered BCG further amplifies immune training, placing the systemic network on enhanced standby. Pre-activated immune cells home to the tumor, and systemically administered ICIs achieve “systemic priming” and “local desensitization.” This establishes a full-chain antitumor network spanning immune cell generation (bone marrow), transport (circulation), and effector function (tumor site).

### 3.5 Enhancing delivery efficiency and intravesical retention

Despite its efficacy in treating NMIBC, intravesical BCG therapy is associated with significant adverse effects, including systemic infection, sepsis, and, in rare cases, death. These safety concerns may lead to treatment discontinuation in some patients, thereby driving the development of safer, non-infectious BCG-derived alternatives such as BCG components. However, compared to these components, live BCG remains more widely used in clinical practice due to its superior immunogenicity. By mimicking a mild natural infection with *Mycobacterium bovis*, live BCG can transiently persist within the host and continuously release a broad spectrum of antigens, thereby inducing more robust and durable cellular immune responses and immune memory, particularly involving central memory T cells and trained immunity (131, 132). In contrast, inactivated BCG components generally exhibit weaker immunogenicity and limited capacity to establish long-term protective immunity. Nevertheless, despite these immunological advantages, the therapeutic efficacy of live BCG is still constrained by rapid urinary washout, insufficient bladder retention, and suboptimal tumor interaction following intravesical instillation. These limitations highlight the necessity of advanced delivery systems (**Table 1**) to improve its stability, localization, and overall therapeutic performance.

**Table 1:** The delivery strategies of BCG.

Delivery strategy	Mechanism	Efficacy	References
Lip-BCG-CWS	1. Enhances the adhesion and internalization of BCG-CWS by bladder cancer cells through positive surface charges and a small particle size; 2. Upregulates the expression of NKG2D ligands, making cancer cells more susceptible to lysis by LAK cells.	A low dose of just 0.1 mg completely inhibited bladder tumor growth and significantly enhanced immune stimulation.	(136–138, 143)
Hydrogel-BCG lysate	1. Upregulate CTSS in APCs to enhance the processing and cross-presentation of tumor-associated antigens; 2. Expand the tumor-specific CD8^+^ T cell receptor repertoire, increase T-cell clonal diversity, induce a pro-inflammatory microenvironment, and promote M1-type macrophage polarization and DC maturation.	It is more effective at inhibiting tumor growth than live BCG; it offers superior antitumor efficacy and safety.	(145, 146)
BCG EVs	The membrane components and contents are similar to those of live microorganisms and can be recognized by the host immune system; they induce bladder cancer cells to produce key cytokines.	Significantly inhibits cancer cell growth and promotes apoptosis *in vitro*; its efficacy *in vivo* requires further validation.	(147–150)
CS NPs-BCG	1. Targets the mucosa via the fibronectin adhesion mechanism, prolonging retention in the bladder and enhancing penetration of the bladder wall; 2. The sustained-release effect maintains a local drug concentration gradient; 3. Chitosan itself acts as a caspase-3 activator, exerting intrinsic antitumor effects and synergizing with BCG.	Exhibits potent antitumor activity without systemic toxicity; significantly improves survival rates in rats	(151–155)
Fe_3_O_4_-BCG	1. Adheres to the bladder in a specific orientation in response to an external magnetic field, preventing it from being washed away by urine; 2. Rapidly forms a porous network gel at body temperature, enabling the encapsulation and controlled release of BCG.	In a rat model, the retention time of BCG in the bladder was significantly extended to 48 hours; this enhanced the local immune response and significantly improved antitumor efficacy.	(157–162)
DOX@BCG	1. Utilize a biotin-streptavidin strategy to anchor the drug and leverage BCG’s specific adhesion to bladder epithelial cells to achieve targeted delivery and prolonged retention; 2. Doxorubicin induces immunogenic cell death, which synergizes with the antitumor immune response triggered by BCG.	It increases local drug concentrations while reducing serum concentrations, thereby minimizing systemic toxicity; it has demonstrated good antitumor efficacy and low side effects in various bladder cancer models.	(170–172, 209)
MBs-BCG	1. With a density lower than that of urine, it floats within the bladder cavity, preventing rapid clearance by urine; 2. The microbubbles gradually degrade, enabling sustained-release of BCG; 3. Supports real-time monitoring via ultrasound imaging.	24-hour bladder retention rate >50%; maintained effective drug concentrations in a simulated urination model; 100% survival rate in rat models, with significant reduction in tumor volume, sustained elevation of cytokines in tissues, and no systemic toxicity.	(174–176)
BCG@MT-A	1. Achieves excellent mucosal adhesion through hydrogen bonding, π-π stacking, and electrostatic interactions; 2. Dissociates and releases its contents in response to the acidic tumor microenvironment (pH 5.5): the release of Mn^2+^ activates the cGAS-STING pathway to promote DC maturation, while the release of AMD3100 blocks the CXCL12/CXCR4 axis to inhibit tumor proliferation and induce immunogenic cell death.	Prolonged bladder retention time; significantly inhibited tumor growth and prolonged survival in a mouse *in situ* bladder cancer model; increased CD8^+^ T cell infiltration, promoted DC maturation and M1 polarization, reduced the proportion of Tregs and PD-L1 expression, with no significant systemic toxicity.	(179–182)

#### 3.5.1 Delivery of BCG-derived components

BCG cell-wall skeleton (BCG-CWS) is the key bioactive component of the vaccine (133). As a ligand for TLR2 and TLR4, it activates APCs and promotes their maturation, driving secretion of TNF-α, IL-6, and IL-12. This triggers robust innate and cellular immune responses, which can be further enhanced by adjuvants. However, its clinical translation remains challenging due to several limitations: poor aqueous solubility with a tendency to aggregate, low cellular uptake efficiency by cancer cells, and the reliance of current formulations on oil-in-water (O/W) emulsification for dispersion, a process often associated with severe inflammatory responses. Furthermore, when administered via Foley catheter for intravesical instillation, it may cause catheter occlusion; additionally, its suboptimal dimensions restrict tissue penetration (134, 135). Encapsulation within nanoparticles can help address these challenges by significantly enhancing dispersibility, stability, targeting capability, and intracellular delivery efficiency. Liposomes, with their high biocompatibility, biodegradability, and ease of modification, represent an ideal drug delivery platform (136, 137). Cationic liposomal systems such as octa-arginine-modified lipid nanoparticles(R8-liposomes) can effectively deliver BCG-CWS(Lip-BCG-CWS) (**Figure 4**). Owing to its positive surface charge and small particle size (~230 nm), this delivery system significantly enhances the binding and endocytosis of the hydrophobic, negatively-charged BCG-CWS by bladder cancer cells, thereby amplifying immune stimulation. Notably, R8-liposomes loaded with just 0.1 mg of BCG-CWS completely suppressed bladder tumor growth, demonstrating superior antitumor efficacy compared to the 1 mg dose group (138). There are three possible mechanisms explaining this non-linear dose-response relationship: First, excessive immune suppression at high doses plays a dominant role. Following the highly efficient delivery of BCG-CWS via R8 liposomes, a large amount of antigen strongly mobilizes MDSCs with immunosuppressive functions. These cells produce excessive NO through IL-1 receptor-dependent pathways, thereby significantly inhibiting T-cell activation and proliferation (139). Concurrently, high-dose BCG antigens can induce a more sustained expansion of Tregs through epigenetic modifications (such as persistent demethylation of the FOXP3 gene), further enhancing the immune-tolerant microenvironment (140). Second, although high-density R8 modification enhances cellular uptake efficiency, the high positive charge density on the liposome surface may excessively mask key glycolipid epitopes (such as mannose residues) on the BCG-CWS surface, interfering with their optimal binding to natural recognition receptors on dendritic cells, such as TLR2 and mannose receptors on dendritic cells, leading to massive internalization of the antigen into non-functional degradation pathways rather than effective cross-presentation pathways (141). Finally, high-dose BCG-CWS may also induce excessive endoplasmic reticulum stress and ROS bursts by overactivating the AMPK/mTOR signaling axis, thereby triggering apoptosis resistance or autophagic death in tumor or immune cells, which weakens the net antitumor effect within the therapeutic window (142). In contrast, low-dose or low-density R8 modification maintains a balance between immune activation and suppression, avoiding excessive NO production, Treg expansion, and antigenic masking, thereby more effectively stimulating antitumor immunity. Emerging evidence suggests that R8-liposome-BCG-CWS can upregulate the expression of NKG2D ligands, thereby sensitizing cancer cells to lysis by lymphokine-activated killer (LAK) cells (143).

**Figure 4:**
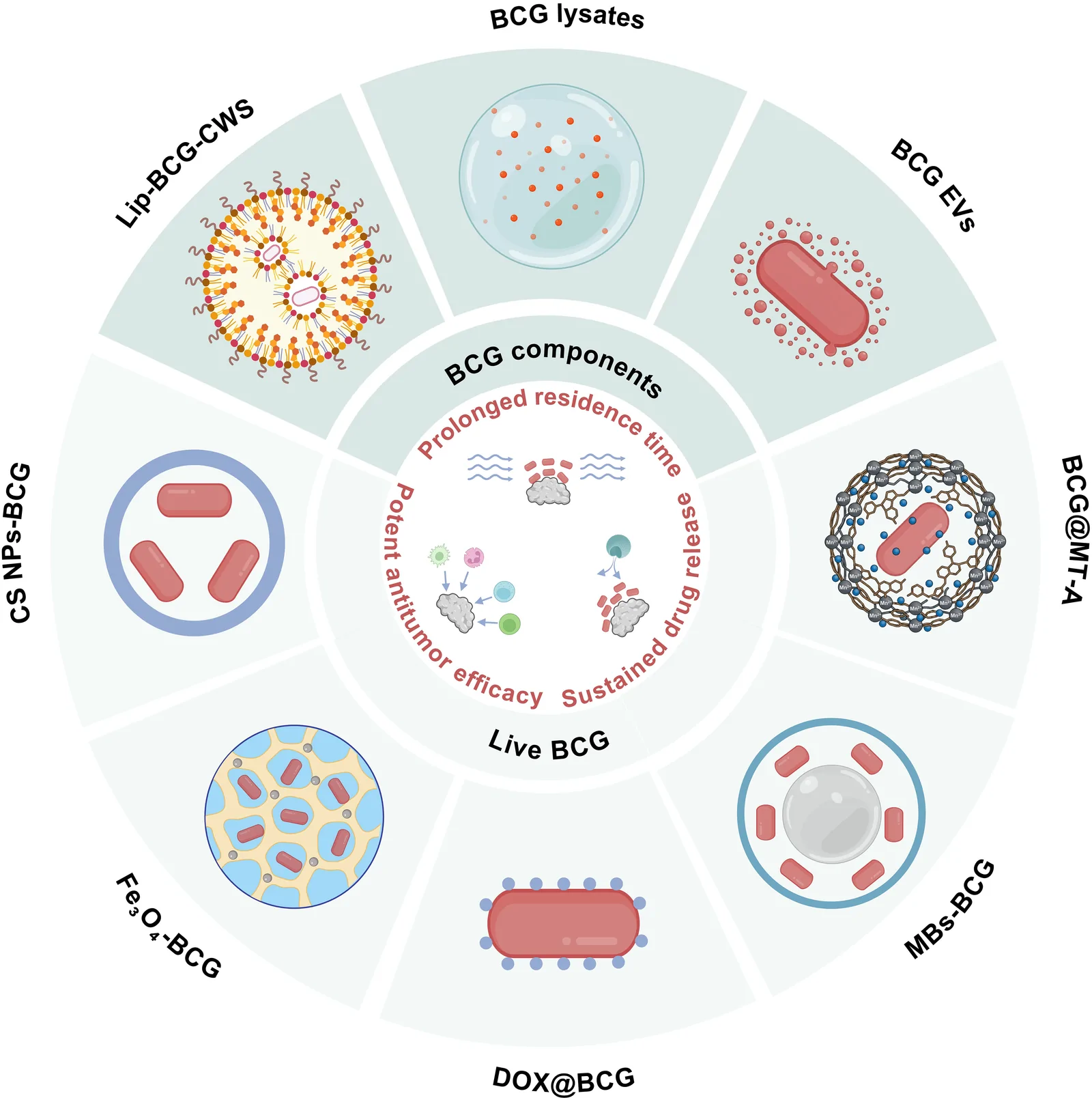
The delivery strategies of BCG. Lip-BCG-CWS enhances cellular uptake through their positive charge and small particle size; BCG lysates and BCG EVs maintain their immunogenicity while also exhibiting a high degree of safety; CS NPs-BCG prolong retention time and enable sustained drug release via their mucosal adhesion properties; Fe_3_O_4_-BCG adhere directionally to the bladder wall under the guidance of an external magnetic field; DOX@BCG achieves targeted delivery and prolonged retention by leveraging BCG’s inherent epithelial adhesion capacity; MBs-BCG floats within the bladder lumen due to their lower density than urine, preventing rapid clearance and enabling sustained release of BCG; BCG@MT-A achieve controlled release through adhesion and dissociation responsive to the tumor’s acidic microenvironment.

Bacterial lysates function as potent non-specific immune stimulants, capable of reprogramming the TME and activating a systemic anti-tumor immune response. Since the discovery of “Coley’s toxins” in the late 19th century, it has been recognized that bacterial lysates can activate the immune system to combat tumor (144). The promising therapeutic effects of BCG lysate have confirmed in melanoma (145, 146). When BCG lysate was loaded onto PLGA-PEG-PLGA hydrogels (**Figure 4**), it was found to be more effective than live BCG in inhibiting tumor growth, inducing a pro-inflammatory TIME, and promoting M1 macrophage polarization and DCs maturation. Mechanistic studies indicate that the BCG hydrogel enhances the processing and cross-presentation of tumor-associated antigens by upregulating cathepsin S (CTSS) in APCs, thereby expanding the tumor-specific CD8^+^ T cell receptor repertoire and increasing T-cell clonal diversity. Although BCG lysate hydrogels have demonstrated superior antitumor efficacy and safety compared to live bacteria, their ability to induce long-term immune memory remains unclear, and batch-to-batch heterogeneity in the lysate may affect the consistency of therapeutic efficacy. Mechanistically, aside from the upregulation of CTSS, it remains to be explored whether the lysate enhances antitumor immunity by modulating trained immunity or through interactions with the bladder microbiota.

Extracellular vesicles (EVs) secreted by prokaryotic and eukaryotic cells have become a hot point of research in the fields of cancer and infectious diseases. Due to their membrane composition and contents resembling those of live microorganisms, EVs can be recognized by the host immune system in a manner similar to live microbes (147, 148). They possess immune-activating and antitumor potential associated with live bacterial therapies while avoiding the infection risks and supply shortages. Current studies show that BCG EVs (**Figure 4**) effectively induce bladder cancer cells to produce key cytokines (such as IL-6, IL-8, and GM-CSF) *in vitro*, significantly suppressing cancer cell growth and promoting apoptosis (149, 150). BCG EVs demonstrate promising antitumor efficacy in *in vitro* models, but their therapeutic effect requires further validation in *in vivo* bladder cancer models. Currently, the mechanism underlying its immune activation remains unclear, and it faces challenges such as low *in vivo* delivery efficiency, difficulties in standardized production, and insufficient long-term stability. Further analysis of the mechanism using animal models is needed, along with the optimization of engineering strategies, to advance clinical translation.

#### 3.5.2 Delivery of live BCG

Chitosan nanoparticles (CS NPs) exhibit favorable mucoadhesive properties. The encapsulation of BCG in chitosan nanoparticles (CS NPs-BCG) (**Figure 4**) significantly prolongs its residence time in the bladder, enhances penetration of the bladder wall, and maintains local drug concentration through a sustained-release effect (151, 152). Furthermore, the residence time of BCG is influenced by the *in vivo* degradation rate of chitosan, which studies have shown is correlated with its degree of deacetylation; a higher degree results in slower degradation (153). This nanocarrier system has two advantages. First, the nanoparticles contain unbound BCG, which can preferentially target the mucosa via the fibronectin adhesion mechanism, rapidly inducing a bladder immune response and delivering a potent initial drug dose. Second, the controlled release of nanoparticle-bound BCG enables sustained concentration gradients at the bladder mucosa, thereby enhancing penetration across the urothelial barrier. Moreover, chitosan itself can exert intrinsic anti-tumor effects by acting as a caspase-3 activator (154), thereby enabling a synergistic action with BCG. *In vivo* results revealed that CS NPs-BCG significantly enhanced rat survival (up to 86 days) and exerted potent anti-tumor effects without systemic toxicity (155).

Currently, various thermosensitive hydrogels are widely used for sustained drug release. Among them, chitosan(CS)/glycerophosphate (GP) hydrogel demonstrates remarkable advantages: First, both the gel and its degradation products are non-toxic (153). Second, its internal pore structure is sufficiently large to accommodate tissue cells (156). Furthermore, its thermosensitive gelation and injectability make it suitable for intravesical instillation. Finally, unlike other thermosensitive hydrogels, CS/GP can be prepared at sub-physiological temperatures, thereby maximizing the preservation of BCG activity (157–160). When incorporated into the CS/GP hydrogel, Fe_3_O_4_ magnetic nanoparticles confer the system (Fe_3_O_4_-BCG) (**Figure 4**) the ability to respond directionally to an external magnetic field, enabling adhesion to the bladder and preventing washout by urine. This magnetic hydrogel rapidly forms a gel at body temperature both *in vitro* and *in vivo*, creating a porous network structure, where BCG is embedded. In a rat model under a 4 kG external magnetic field, the gel system significantly prolonged BCG retention in the bladder to 48 hours, enhanced local immune responses and markedly improved anti-tumor efficacy (161). However, the initial burst release of BCG from this hydrogel may contribute to treatment-related side effects. Strategies to mitigate this include using high-molecular-weight chitosan, increasing the hydrogel-to-BCG ratio, or applying surface modifications and polymer coatings. These approaches reduce the hydrogel’s surface porosity, thereby minimizing burst release and improving patient tolerance to intravesical BCG therapy (162). Although few studies have investigated the long-term effects of static magnetic fields on human peribladder tissues, similar magnetic therapies have been widely used in clinical practice for the treatment of female urinary incontinence. Relevant studies have confirmed their high safety profile, with only about 13% of patients experiencing transient reactions such as diarrhea, muscle pain, and limb discomfort, and none discontinuing treatment due to intolerance (163). Furthermore, magnetothermy—a therapy that utilizes external alternating magnetic fields to activate magnetic nanoparticles delivered to tumor sites, thereby generating a controlled local thermal effect—is gaining traction in the field of anticancer therapy. Currently employed as an adjunct to radiation therapy, this approach has been shown to significantly improve the prognosis of glioma patients (164, 165). Beyond brain tumors, magnetothermy has also demonstrated promising results in the treatment of pancreatic cancer, breast cancer, and peritoneal metastases (166–168). These developments highlight the future clinical translation potential of Fe_3_O_4_-BCG therapy for NMIBC. However, potential challenges cannot be overlooked. This therapy typically requires the continuous application of a directed static magnetic field after instillation to maintain the adhesion of magnetic particles to the bladder wall; yet, in clinical bladder instillation scenarios, patients must remain in a dedicated magnetic confinement device for an extended period after instillation, resulting in restricted mobility. To address this issue, researchers have attempted to design portable external magnetic field devices that use strong external magnetic fields to attract drug-loaded magnetic particles to the bladder wall, achieving sustained drug release in animal models (169). These results indicate that, through the rational design of external magnetic confinement devices, the clinical feasibility of directed magnetic fields is not insurmountable. However, since abdominal skin and fat thickness vary among individuals, and urination during treatment alters the shape of the bladder and its relationship with the magnetic field, further engineering challenges must be addressed to facilitate the transition from animal experiments to human applications. These include device adaptation, individualized calibration of magnetic field strength, and design considerations for compatibility with the physiological act of urination.

Beyond its direct therapeutic effects, BCG is being explored as a novel delivery carrier for transporting and releasing other drug molecules. Recently, a live BCG-based drug delivery system (DOX@BCG) (**Figure 4**) has garnered significant attention. This system utilizes a biotin–streptavidin strategy to stably anchor poly (lactic-co-glycolic acid) (PLGA) nanoparticles loaded with the chemotherapeutic drug doxorubicin (DOX) onto the surface of BCG (170). Leveraging BCG’s specific adhesion capability to bladder epithelium, DOX@BCG enables targeted delivery and prolonged retention of the drug at tumor sites, thereby enhancing local drug concentration while reducing systemic toxicity. Although DOX@BCG and WT-BCG show no significant differences in stimulating cytokine production in PBMCs or in activating TLR, DOX contributes an additional mechanism: it enters tumor cells to induce immunogenic cell death and trigger anti-tumor immune responses, synergizing with BCG. In multiple bladder cancer models, DOX@BCG demonstrated favorable antitumor efficacy with attenuated systemic side effects. This improved safety may be attributed to the preferential accumulation of DOX@BCG within the bladder, leading to lower serum concentrations (170–172).

Current strategies for intravesical BCG delivery primarily rely on adhesive nanosystems and external magnetic field-responsive systems. However, nanoparticles adhering to the bladder epithelium may disrupt normal physiological function, while the requirement for specialized equipment limits the use of magnetic systems (173). Microbubbles are widely used as agents in ultrasound imaging. Recently, their application as drug delivery vectors has gained significant attention due to their dual advantages in tumor diagnosis and therapy (174, 175). An air-filled microbubble-based drug delivery system (MBs-BCG) (**Figure 4**) has been developed to offer a promising solution to these challenges mentioned above. The MBs-BCG comprises an air core, an intermediate solution layer, and an outer shell. This design enables efficient encapsulation of viable BCG with a bacterial loading rate reaching 25%. Unlike conventional nanocarriers that adhere to the urothelium, MBs-BCG float within the bladder lumen due to their lower overall density compared to urine, thereby avoiding rapid clearance. The bladder retention rate of MBs-BCG exceeds 50% within 24 hours. This is due to the use of highly cross-linked bovine serum albumin (BSA) as a gas permeation barrier in the shell. Conventional lipid shells or uncross-linked protein shell micelles have porous shells and high gas permeability; driven by Laplace pressure, the internal air rapidly diffuses through the shell into the surrounding liquid, causing the micelles to dissolve within minutes to hours (i.e., Ostwald ripening). In contrast, MBs-BCG form a three-dimensional, covalently cross-linked, dense protein network through an interfacial polymerization reaction between phthaloyl chloride and the amino groups of BSA. This cross-linked structure not only significantly reduces the diffusion coefficient of gas molecules within the shell but also increases the shell thickness to approximately 8.5 micrometers, further extending the physical path of gas diffusion. At the same time, the cross-linking confers a high elastic modulus and mechanical rigidity to the shell, enabling it to resist deformation or collapse caused by changes in internal air pressure, thereby maintaining a stable gas-liquid interface. Furthermore, the freeze-drying and rehydration steps during the preparation process pre-form stable “gas traps” within the microbubbles—cavities left by water sublimation during freeze-drying, into which liquid infiltrates during resuspension while trapping air within the core. This structure prevents rapid dissolution triggered by interfacial tension acting directly on the tiny gas nuclei. Taken together, the cross-linked BSA shell of BCG-MBs acts as an efficient, dense, and low-permeability gas barrier, extending the dissolution time of the air core from the typical few minutes to several days. Within the bladder, the microbubbles gradually degrade, enabling sustained BCG release and maintaining effective local drug concentrations. In a simulated periodic-voiding model, BCG concentrations remained above therapeutic levels throughout both day and night, sufficient to elicit a robust immune response. MBs-BCG demonstrated significant anti-tumor efficacy in a rat bladder cancer model. Tumor volume was markedly reduced, survival reached 100%, and elevated levels of cytokines such as IL-2, IFN-γ, and TNF-α were sustained in bladder tissues. No systemic toxicity was observed. Additionally, this system allows real-time monitoring via ultrasound imaging, offering a promising strategy for personalized and precision treatment in bladder cancer patients (176). Although the MBs-BCG system avoids mucosal damage and rapid clearance through sustained-release via flotation, the stability of its air core and its degradation rate *in vivo* still need to be explored, as these factors may affect the precise control of drug release.

Metal-phenolic networks (MPNs) exhibit excellent mucosal adhesion properties, which stem from hydrogen bonding, π-π stacking, and electrostatic interactions between their catechol groups and mucosal proteins. Furthermore, this coating undergoes responsive dissociation in the acidic TME (pH 5.5), thereby enabling controlled drug release (177, 178). Encapsulating BCG within MPNs assembled from Mn^2+^and tannic acid, and further loading them with AMD3100 (BCG@MT-A) (179) (**Figure 4**), significantly prolonged their retention time in the bladder. By releasing Mn^2+^ and AMD3100 in response to the tumor’s acidic microenvironment, respectively activating the cGAS-Sting pathway to promote DCs maturation and blocking the CXCL12/CXCR4 axis to directly inhibit tumor proliferation and induce immunogenic cell death (180–182). *In vivo* results demonstrated that BCG@MT-A significantly inhibited tumor growth in a mouse model of *in situ* bladder cancer, prolonged survival, and effectively increased CD8^+^ T cell infiltration, promoted DC maturation and M1-type macrophage polarization, while simultaneously reducing the proportion of regulatory T cells and PD-L1 expression, without causing significant systemic toxicity (179). Although BCG@MT-A significantly inhibits bladder cancer due to MPN’s mucosal adhesion and acid-responsive drug release properties, the potential neurotoxicity and long-term safety of Mn^2+^ still need to be verified, and the impact of dynamic pH fluctuations within the bladder on the drug’s release behavior also requires further evaluation.

## 4 The relationship between BCG and the bladder microbiome

Traditionally, the bladder was considered a sterile environment in the absence of infection. However, recent microbiome studies based on high-throughput sequencing have completely overturned this view, confirming the presence of a complex and highly variable microbial community within the bladder, known as the “urinary microbiome.” This discovery offers a new perspective for re-examining the mechanism of action of BCG. As an attenuated live bacterium, the therapeutic efficacy of BCG is highly dependent on its effective colonization and survival on the surface of the bladder mucosa, as well as subsequent interactions with the uroepithelium and the immune system. Theoretically, following BCG bladder instillation, BCG can influence the urinary microbiome, and conversely, the urinary microbiome can influence BCG. Therefore, understanding the relationship between BCG and the urinary microbiome is of critical importance.

### 4.1 The bladder microbiome regulates BCG colonization and internalization

The tumor microbiome of the bladder may alter the response of NMIBC to BCG immunotherapy by modulating the local immune environment and influencing BCG’s ability to colonize. Specifically, 16S sequencing and metagenomic analysis revealed significant differences in tumor microbiome composition between BCG responders and non-responders, with Lactobacillus spp. being significantly enriched in responders. Further *in vitro* co-culture experiments demonstrated that Lactobacillus crispatus significantly enhances BCG internalization in urothelial carcinoma cells in a dose-dependent manner, suggesting that specific commensal bacteria may enhance the immunotherapeutic efficacy of BCG by facilitating its entry into tumor cells (183, 184). These findings indicate that the bladder microbiome, particularly certain lactic acid-producing bacteria, may modulate the overall efficacy of BCG therapy by influencing BCG colonization and tumor cell internalization.

### 4.2 BCG modulates the bladder microbiome

Intravesical instillation of BCG exerts a significant downregulating effect on the local bladder microbiota. Specifically, BCG treatment significantly reduced the α-diversity (particularly richness) of the bladder microbiota in tumor-bearing mice. Compared with untreated or healthy control groups, BCG treatment caused the previously dominant Enterococcaceae to almost completely disappear from the bladders of tumor-bearing mice, while significantly enriching bacterial groups such as Staphylococcaceae and Moraxellaceae. Furthermore, BCG treatment suppressed the relative abundance of potential pathogenic genera associated with bladder tumors, such as Escherichia/Shigella and Micrococcus. These results suggest that BCG may reshape the composition and structure of the bladder microbiota through competitive exclusion, induction of local immune responses, or alteration of the bladder mucosal microenvironment, thereby “reversing” the ecological advantage of tumor-associated bacterial communities and indirectly enhancing the antitumor immune response (185).

### 4.3 Microbiome composition is associated with the BCG immune response and clinical prognosis

Changes in urinary tract microbiota—specifically the abundance and diversity of certain bacterial genera—are closely linked to BCG immunotherapy response and clinical outcomes in NMIBC patients. Before treatment, the genus Aureispira was 12-fold more abundant in BCG non-responders than responders, while Negativicoccus succinicivorans was 27-fold less abundant, suggesting these microbes as potential biomarkers for treatment resistance. Non-responders were dominated by Lactobacillus, Escherichia–Shigella, and Clostridia, whereas responders showed more Enterococcus, Serratia, and Lactobacillus. During BCG therapy, Aureispira in non-responders gradually declined to one-eighth of baseline by treatment end, while Nitrosospira, Parabacteroides johnsonii, and the family Pirellulaceae significantly increased (186). Clinical studies show that oral Lactobacillus casei reduces bladder cancer recurrence risk, possibly by enhancing local Th1-type immunity and inducing antitumor cytotoxicity. Moreover, recurrence-free patients had higher urinary abundance of Lactobacillales, while those with recurrence were dominated by Enterobacteriales (187). Thus, differences in specific genera (e.g., Aureispira, N. succinicivorans, Lactobacillus) and dynamic microbial shifts not only reflect BCG immune response status but also hold promise as novel biomarkers and therapeutic targets for predicting treatment response and prognosis.

## 5 Discussion

Bladder cancer, particularly high-risk NMIBC, poses a long-standing clinical challenge due to its high rates of recurrence and disease progression (188, 189). As a first-line treatment for NMIBC, BCG immunotherapy activates a multi-layered immune response (190). However, the limited efficacy and side effects of BCG monotherapy have prompted researchers to explore engineered strategies to enhance its therapeutic capacity (191). The diverse bioengineering strategies summarized in this review including genetic modification, immune potentiation, and advanced delivery systems, have significantly expanded the therapeutic potential of BCG. However, these approaches are often developed in isolation, lacking a unifying conceptual framework that connects engineering design with the underlying immunological processes. Here, we propose a conceptual multi-layered framework for BCG immunotherapy, in which therapeutic efficacy is governed by coordinated regulation across four functional layers—initiation, amplification, shaping, and maintenance. This framework shifts current efforts from empirical optimization toward mechanism-guided and system-level engineering strategies.

### 5.1 A multi-layered framework for BCG immunotherapy

BCG-induced antitumor immunity is a dynamic and coordinated process that can be broadly divided into four functional layers: initiation, amplification, shaping, and maintenance (**Table 2**). The initiation phase involves bacterial adhesion, internalization, and antigen release, which determine the efficiency of tumor–BCG interaction and subsequent antigen presentation. The amplification phase is characterized by the activation of innate immune pathways, including STING signaling and metabolic reprogramming associated with trained immunity. The shaping phase involves the orchestration of cytokine networks and immune cell interactions within the TIME, while the maintenance phase supports long-term immune surveillance through memory T cells and reprogrammed myeloid populations. Most current engineering strategies target only a single layer of this process. For example, FimH-based modifications primarily enhance the initiation phase (80), while disA-overexpressing BCG strains amplify innate signaling (108). Similarly, cytokine-secreting recombinant BCG strains reshape the TIME but may not address upstream limitations in antigen presentation or downstream deficits in immune memory (119–124). This fragmentation may partly explain the inconsistent therapeutic outcomes observed across studies. Therefore, future BCG engineering should prioritize cross-layer integration, in which multiple immunological processes are co-optimized to achieve synergistic and durable antitumor responses.

**Table 2:** Mapping of engineering strategies to the four functional layers.

Chapters	Strategies	Functional layers
3.1.1	rBCG-S. FimH	Initiation
3.1.2	rBCG-SIC-dltA	Amplification
3.1.3	VPM1002	
3.2.1	rBCG-disA-OE	
3.2.2	rBCG-S1PT/LTK63	
3.3.1	rBCG-Cytokine	Shaping
3.5	Delivery of BCG-derived components or live BCG	Maintenance

### 5.2 Revisiting the role of type 2 immunity in BCG therapy

A large body of existing clinical and mechanistic evidence continues to consistently indicate that the therapeutic efficacy of intravesical BCG is centered on inducing Th1-type immunity: BCG instillation enriches the urinary cytokine microenvironment with inflammatory mediators such as IL-2, IL-12, IFN-γ, TNF-α, and IL-18 (192), among which IFN-γ and IL-12 are essential for BCG-mediated tumor control in orthotopic bladder cancer models (193), IL-10 deficiency can enhance efficacy (194), and CD4^+^ and CD8^+^ T cells, together with tumor-intrinsic IFN-γ receptor signaling, jointly participate in effective tumor clearance (193, 195). Clinical cohorts also show that a highly enriched Th1/Th17-like immune landscape predicts prolonged recurrence-free survival after BCG treatment (26). However, emerging evidence suggests that type 2 immunity is not simply an antagonist of type 1 immunity, but rather acts as a “shaping layer” that influences BCG efficacy by regulating cytokine network balance and T cell differentiation fate, rather than participating in initial bacterial recognition or long-term memory maintenance. During BCG treatment, type 2-related pathways are often co-activated, for example, induction of IL-4 in the rBCG-IFN-γ-treated environment and upregulation of IL-10 and IL-6 in toxin-engineered strains; IL-4 may exert a complementary effect by limiting terminal exhaustion and maintaining T cell function (196), while simultaneously promoting myeloid cell reprogramming (63). Although the routes of administration differ, this conceptual finding provides important insights into how cytokines regulate the differentiation state of T cells. More directly, a study using intravesical infusion of the IL-4-SA fusion protein confirmed that, in a mouse model of superficial bladder cancer, IL-4-SA can stably anchor to the bladder mucosa for up to 5 days, inducing significant CD8^+^ T-cell infiltration and tumor-specific CTL activity (reaching 31.0% ± 3.0% at an effector-to-target ratio of 50:1), resulting in long-term tumor-free survival in 50% of tumor-bearing mice (197). Nevertheless, the evidence supporting a synergistic role of Th2 currently comes mostly from small-scale preliminary studies, retrospective/correlational analyses, and *in vitro* or preclinical models, and the role of GATA3 in bladder cancer progression is also highly context-dependent and even contradictory (198, 199). Therefore, a more reasonable positioning is that type 1 immunity remains the generally validated and indispensable cornerstone of the antitumor effects of BCG, whereas IL-4/type 2 immunity should be considered a potential regulatory or predictive factor rather than a primary effector mechanism. On this basis, future bioengineering strategies should, while strengthening durable type 1 antitumor immunity, longitudinally analyze type 2-related markers and explore rational combinations that can dynamically balance type 1 and type 2 immunity; although excessive type 1 dominance can drive acute tumor killing, it may accelerate T cell exhaustion and tissue damage, whereas appropriate type 2 participation may help promote immune durability and tissue repair, thereby improving the overall efficacy of BCG immunotherapy.

### 5.3 BCG as a dynamic and programmable immunological system

Conventional engineering approaches often treat BCG as a static delivery vehicle for antigens or immunomodulatory molecules. However, as a live attenuated organism, BCG represents a dynamic and self-regulating immunological system capable of interacting with the host environment in real time. This unique property offers opportunities for developing next-generation programmable BCG platforms. One promising direction is the incorporation of environmentally responsive genetic circuits that enable BCG to modulate its activity in response to tumor-specific cues, such as hypoxia, pH, or inflammatory signals (200). In addition, modular engineering strategies that combine multiple functional elements such as adhesion enhancement, innate immune activation, and cytokine secretion could enable coordinated regulation across different stages of the immune response. Temporal control of these functions may further optimize therapeutic outcomes by aligning immune activation with disease progression, for example, by prioritizing innate activation during early treatment and promoting immune memory during later stages. Alternatively, future engineered BCGs will integrate the four dimensions of “initiation,” “amplification,” “shaping,” and “maintenance,” thereby not only enhancing adhesion but also stimulating the host’s immune response, reshaping the immune microenvironment, and ensuring long-lasting therapeutic effects.

### 5.4 Active roles of delivery systems in immunomodulation

Advanced delivery systems have been primarily developed to address the pharmacokinetic limitations of intravesical BCG, including rapid washout and limited bladder retention. They primarily support the maintenance layer by sustaining long-term immune stimulation; they can also contribute to the initiation layer and amplification layer by improving bacterial retention and initial antigen availability. While these systems significantly improve local drug availability, their potential roles in modulating immune responses remain underexplored. We propose that next-generation delivery platforms should not be viewed as passive carriers but as active participants in immunotherapy. For example, biomaterials such as hydrogels and nanoparticles may influence local immune activation, antigen presentation, and myeloid cell polarization through their physicochemical properties (201). Similarly, microbubble-based systems offer unique opportunities for spatial and temporal control of BCG distribution within the bladder. Integrating immunomodulatory functions into delivery systems could therefore enhance both the magnitude and durability of BCG-induced immune responses.

### 5.5 Toward precision BCG immunotherapy

Despite its clinical success, approximately half of patients with NMIBC fail to achieve durable responses to BCG therapy. This highlights the urgent need for patient stratification and precision treatment strategies. Biomarker-guided approaches may help identify patients who are most likely to benefit from specific engineering strategies and predict patient prognosis. For instance, tumor expression of mannose residues may predict responsiveness to adhesion-enhanced BCG strains (80), while baseline activation of the STING pathway or immune subtype classification may inform the selection of innate immune modulator (105). Recent multi-omics studies confirm that integrating stratification biomarkers—such as tumor mutational burden (TMB), PD-L1 expression dynamics, and urinary microbiome characteristics—can more accurately help determine the prognosis of patients receiving BCG therapy. Specifically, TMB assesses the potential for neoantigen generation by quantifying the number of somatic mutations. A high TMB enhances BCG-induced T-cell recognition and is associated with patient benefit from BCG immunotherapy; however, its predictive power is limited by the integrity of antigen presentation pathways—if presentation defects exist, even a high TMB cannot effectively activate antitumor immunity (202, 203); Currently, multiple studies have confirmed that elevated PD-L1 expression levels following BCG treatment are associated with a better clinical prognosis, suggesting that it reflects the initiation of effective immune surveillance. However, some viewpoints suggest that persistently high levels of PD-L1 expression may lead to T-cell exhaustion, thereby limiting the long-term efficacy of BCG; Therefore, PD-L1 serves not only as a static stratification marker but also as a key window for dynamically monitoring the remodeling of the immune microenvironment, with urinary PD-L1 testing offering the possibility of non-invasive, real-time monitoring (204–207); As a live bacterial preparation, BCG’s colonization and immune activation within the bladder are significantly influenced by the resident microbiota. Studies have found significant differences in baseline microbial composition between responders and non-responders; for example, the abundance of Lactobacillus is positively correlated with BCG efficacy, while the overgrowth of opportunistic pathogens may impair efficacy. These findings suggest that assessing urinary microbiome characteristics prior to treatment—or even modulating the microbiome through pre-treatment with probiotics or antibiotics—may represent a new strategy to improve BCG response rates (186, 208). In addition, combination therapies involving BCG and immune checkpoint inhibitors, chemotherapy, or cytokine modulation represent promising avenues for improving outcomes in BCG-unresponsive patients. Importantly, such combinations should be guided by mechanistic insights rather than empirical testing, with careful consideration of immune dynamics and potential synergistic interactions.

### 5.6 Conclusions and future perspectives

In summary, the next phase of BCG immunotherapy development will require a paradigm shift from single-factor optimization to integrated, mechanism-driven design. Key priorities include the development of multi-layered engineering strategies, the incorporation of both type 1 and type 2 immune modulation, the design of programmable BCG systems, and the implementation of precision medicine approaches. By aligning bioengineering innovations with a deeper understanding of immune regulation, it may be possible to unlock the full therapeutic potential of BCG and establish more effective and durable treatments for bladder cancer.

## Glossary

NMIBC: Non-muscle-invasive bladder cancer
BCG: Bacillus Calmette–Guerin
GAG: Glycosaminoglycan
FAP: Fibronectin attachment protein
FN: Fibronectin
Rac1: Ras-related C3 botulinum toxin substrate 1
Cdc42: Cell division control protein 42
Pak1: p21-activated kinase 1
PTEN: Phosphatase and tensin homolog deleted on chromosome ten
IRAK: Interleukin-1 receptor associated kinase
TLR: Toll like receptor
HMGB1: High mobility group box-1 protein
APCs: Antigen-presenting cells
iNOS: Inducible nitric oxide synthase
ICD: Immunogenic cell death
NO: Nitric oxide
PAMPs: Pathogen-associated molecular patterns
PRRs: Pattern recognition receptors
MARCH1: Membrane-associated RING-CH 1
NK cells: Natural killer cells
MAIT cells: Mucosal-associated invariant T cells
TME: Tumor microenvironment
Cx43: Connexin 43
PBMCs: Peripheral blood mononuclear cells
KIRs: Killer-cell immunoglobulin-like receptors
ADCC: Antibody-dependent cellular cytotoxicity
NKG2D: Natural killer group 2 member D
NETs: Neutrophil extracellular traps
TRAIL: TNF-related apoptosis-inducing ligand
DCs: Dendritic cells
Bcl-2: B-cell lymphoma-2
Bcl-xL: B-cell lymphoma-extra large
ESAT6: Antigenic target of 6 kDa
CFP10: Culture filtrate protein 10
HSPCs: Hematopoietic stem and progenitor cells
SNPs: Single nucleotide polymorphisms
IRF: Interferon regulatory factor
STAT: Signal transducer and activator of transcription
ROS: Reactive oxygen species
FLT3LG: Fms-like tyrosine kinase 3 ligand
MDSC: Myeloid-derived suppressor cell
c-di-AMP: Cyclic di-AMP
disA: Diadenylate cyclase gene
GLUT1: Glucose transporter 1
WT-BCG: Wild-type BCG
SIC: Streptococcal inhibitor of complement
dltA: D-alanyl carrier protein ligase
S1PT: S1 subunit of pertussis toxin
FC: Fragment C
LTAK63: The non-toxic A subunit of the heat-labile toxin
ESX-1: ESAT-6 secretion system-1
ureC: The urease C gene
hly: The listeriolysin O encoding gene (hly)
LLO: Listeriolysin O
ICIs: Immune checkpoint inhibitors
BCG-CWS: BCG cell-wall skeleton
CTSS: Cathepsin S
O/W: Oil-in-water
EVs: Extracellular vesicles
LAK: Lymphokine-activated killer
CS: Chitosan
GP: Glycerophosphate
PLGA: Poly (lactic-co-glycolic acid)
LNP: Lipid nanoparticle
MBs: Microbubbles
MPNs: Metal-phenolic networks
TMB: Tumor mutational burden
